# Food Inhibits the Oral Bioavailability of the Major Green Tea Antioxidant Epigallocatechin Gallate in Humans

**DOI:** 10.3390/antiox4020373

**Published:** 2015-05-27

**Authors:** Nenad Naumovski, Barbara L. Blades, Paul D. Roach

**Affiliations:** 1 School of Public Health and Nutrition, University of Canberra, Canberra 2601, ACT, Australia; 2 School of Environmental & Life Sciences, University of Newcastle, Ourimbah 2258, NSW, Australia; E-Mails: Barbara.blades@newcastle.edu.au (B.L.B.); Paul.Roach@newcastle.edu.au (P.D.R.)

**Keywords:** EGCG, systemic absorption, green tea catechins, functional foods

## Abstract

The bioavailability of the most abundant and most active green tea antioxidant, epigallocatechin gallate (EGCG) remains uncertain. Therefore, the systemic absorption of EGCG was tested in healthy fasted humans. It was administered as capsules with water or with a light breakfast, or when incorporated within a strawberry sorbet. The results for plasma EGCG clearly revealed that taking EGCG capsules without food was better; the AUC was 2.7 and 3.9 times higher than when EGCG capsules were taken with a light breakfast (*p* = 0.044) or with EGCG imbedded in the strawberry sorbet (*p* = 0.019), respectively. This pattern was also observed for C_max_ and C_av_. Therefore, ingesting food at the same time as EGCG, whether it was imbedded or not in food, substantially inhibited the absorption of the catechin. As with some types of medications that are affected by food, it appears that EGCG should be taken without food in order to maximise its systemic absorption. Therefore, based on these findings, ingesting EGCG with water on an empty stomach is the most appropriate method for the oral delivery of EGCG in clinical trials where EGCG is to be investigated as a potential bioactive nutraceutical in humans.

## 1. Introduction

Green tea (*Camellia sinensis*) is one of the oldest beverages known to man in that it has been consumed for thousands of years. In recent times, an increased consumption of green tea has been linked with a wide range of different health benefits such as an increase in antioxidative potential [1,2,3,4], a reduction in the mortality rate from cardiovascular disease [5,6,7,8,9], a reduced development of coronary artery disease [10] and a lowering of plasma cholesterol [11]. Furthermore, there has been a rise in research on the individual green tea components, particularly its major component epigallocatechin gallate (EGCG), as attractive targets for the development of nutraceuticals [12,13] and functional foods [14,15,16].

As the most abundant green tea (GT) constituent, EGCG has been the focus of research in relation to the reduction of morbidity and mortality as a consequence of cardiovascular disease. Several mechanisms of action have been proposed as to why EGCG may benefit cardiovascular health in humans, such as the lowering of plasma cholesterol through a reduction of cholesterol absorption [17], a decrease in cholesterol synthesis [18] and/or an increase in the cholesterol clearance rate through an upregulation of the LDL receptor [19,20,21,22]. However, many of the studies of this type in humans have used hot/cold GT preparations or whole GT extracts in powder form that considerably vary in their content of EGCG, other catechins and other components naturally found in GT such as caffeine [18,21,23,24,25]. Therefore, despite the fact that EGCG is the predominant compound found in these preparations it is still uncertain whether pure EGCG would exhibit the same health properties in humans as EGCG in a polyphenolic matrix such as GT or GT extracts.

The relatively recent availability of pure, crystalline, stable and affordable EGCG has enabled the design of studies with controlled amounts of EGCG [26,27,28]. Consequently, capsules containing precisely measured amounts of EGCG can be utilised in controlled clinical trials and measured amounts of the crystalline form of EGCG can be incorporated into food products for use as nutraceuticals or functional foods. However, incorporating EGCG into food products requires dissolving the crystalline form in various liquids which could affect the stability of the EGCG and potentially lead to its degradation [29,30,31,32].

The processing temperature and pH of EGCG solutions can influence the catechin’s structural stability and cause degradation and epimerization [31]. Although these processes have been observed to mainly occur at high temperatures and high pH, significant degradation of resolubilised EGCG has also been reported at temperatures below 25 °C [33,34]. Therefore, to minimise the loss of EGCG, solutions need to be made at pH below 4 [34] and kept at low temperatures prior to being used. The use of reducing agents such as ascorbic acid [35] can also be useful.

The modification and use of foods in order to deliver beneficial health outcomes, commonly referred to as functional foods, is a fast growing area of research [36]. Apart from the health benefits that these functional foods can bring, the delivery of a functional component within a food can increase the convenience of the component’s consumption and may also reduce the fasting time. A recent study by Hirun and Roach [37] found that EGCG imbedded in a strawberry sorbet had very good stability during frozen storage for at least 4 months, a benefit ascribed to the low temperature and pH. In addition, this study [37] and a study by Green *et al.* [38] showed that the stability of EGCG under simulated digestion conditions increased when GT was in the strawberry sorbet or mixed with fruit juices, respectively. However, there are no reports on the effectiveness of EGCG once it is incorporated into a food product relative to any health outcomes in humans.

Irrespective of the format in which it is ingested, EGCG needs to be bioavailable in order to have health outcomes [39]. Studies on the bioavailability of pure EGCG in humans are limited [27,40,41] as such studies have primarily focused on the pharmacokinetic properties of preparations where EGCG was imbedded in a polyphenolic complex, as part of GT extracts [24,40,41,42]. In one study, the oral administration of pure EGCG at a dose of 1.6 g in healthy human volunteers [27] produced physiologically-relevant plasma EGCG concentrations (greater than 1 μmol/L) capable of having beneficial health effects. Although there were variations between individuals, the peak EGCG concentrations were reached between 1.3 and 2.2 h after ingestion and the mean elimination half-life ranged from 1.9 to 4.6 h. However, in this study, only encapsulated EGCG was studied as the method of delivery and only after a 10 h fast [27].

To date, only one study in humans has examined the oral bioavailability of EGCG, provided with and without food (a light breakfast) and the EGCG was imbedded in a GT extract [42]. The findings of this study indicated that EGCG (400 and 800 mg), when taken in the form of a polyphenolic complex, showed a greater systemic absorption when the extract was taken on an empty stomach after an overnight fast and it was very well tolerated with only mild symptoms of discomfort reported [42].

Based on the findings of the previous studies [24,27,40,41,42] the present study aimed to determine whether the systemic absorption of pure EGCG would be similarly decreased by the presence of food in the form of a light breakfast, as it was for EGCG in the complex polyphenolic GT extract [42]. Another aim was to test whether EGCG imbedded in a low pH food product such as a strawberry sorbet, could enhance the absorption of EGCG. Therefore, the systemic absorption of EGCG was tested in healthy human volunteers after an overnight fast with the EGCG administered by itself within capsules without breakfast, in capsules taken with a light breakfast or incorporated within a strawberry sorbet, to determine which of the three methods of delivery was the most effective.

## 2. Experimental Section

### 2.1. Materials and Reagents

The EGCG (Teavigo, DSM Nutritionals, Heerlen, The Netherlands) was purchased from RejuvaCare International Pty Ltd. (Sydney, NSW, Australia). All chemicals, (+)-catechin, (−)-epigallocatechin gallate (EGCG), ascorbic acid, disodium ethylene tetra acetate (EDTA), potassium dihydrogen phosphate (KH_2_PO_4_), formic acid, acetonitrile, ethyl acetate, 4-aminosalycilic acid and carboxyl methyl cellulose were purchased from Sigma-Aldrich (Castle Hill, NSW, Australia). Ultrapure deionized water was prepared on the day using a Millipore Milli-Q water purification system (Millipore Australia, North Ryde, NSW, Australia). Gelatin capsule casings were purchased from Melbourne Food Ingredient Depot (Melbourne, VIC, Australia), whey protein isolate was purchased from Vital Strength Nutraceuticals (Marrickville, NSW, Australia) while all food ingredients were purchased from local supermarkets.

#### Preparation and Quality Control of EGCG Products for Oral Delivery

The concentration (purity) of the EGCG in the Teavigo was stated by the manufacturer (DSM Nutritionals, Heerlen, The Netherlands) to be at least 92% (w/w) EGCG. However, in our hands, the powder was measured by HPLC to be 100% ± 2% (w/w) EGCG using EGCG purchased from Sigma-Aldrich (Castle Hill, NSW, Australia) as the external standard, taking into account that the EGCG from Sigma is 97% pure. Therefore, the Teavigo was taken to be 100% EGCG in this study.

The clear gelatin capsule casings were filled with EGCG using a portable capsule filler (Cap-M-Quick, Murietta, USA) to 250 ± 5 mg and capsules showing a variation greater than 2.5% from the target weight were discarded and not used in the study.

The strawberry sorbet was prepared based on a previously published method [37] with the ingredients shown in Table 1. However, as the preparation of the strawberry sorbet required solubilisation in an aqueous medium, heating and exposure to light, conditions that are not favorable for EGCG [31,33,34], the amount of EGCG left in the sorbet after preparation was determined. Briefly, 100 mL of methanol, containing 100 mmol/L 4-aminosalycilic acid solution as an internal standard, was added to 50 g of strawberry sorbet thawed at 4 °C and was incubated on a shaking water bath (Ratek Instruments, Boronia, VIC, Australia) at 60 °C for 1 h and samples were filtered under vacuum through a Whatman No. 1 filter paper (Sigma-Aldrich, Castle Hill, NSW, Australia), passed through a 0.45 μm syringe nylon filter (Phenomenex, Pennant Hills, NSW, Australia) and injected onto a high pressure liquid chromatography (HPLC) system for analysis as described previously [43,44].

**Table 1:** Ingredients used in the preparation of the strawberry sorbet.

Ingredient	Weight (g)	% (w/w)
Strawberries (Creative Gourmet Pty Ltd., Silverwater, NSW, Australia)	250	76.63
Caster Sugar (CSR, Yarraville, VIC, Australia)	45	13.79
Strawberry Flavoured WPI (Vital Strength Nutraceuticals, Marrickville, NSW, Australia)	30	9.20
Carboxyl methyl cellulose (Sigma-Aldrich, Castle Hill, NSW, Australia)	0.40	0.12
EGCG (RejuvaCare International, Sydney, NSW, Australia)	0.84	0.25
Total Weight	326.24	100

The extraction was done in duplicate on five different storage containers of strawberry sorbet with EGCG and on one storage container of strawberry sorbet prepared without EGCG (control). To test for the presence of peaks in the sorbet, which could interfere with the 4-aminosalycilic acid internal standard peak as well as with the EGCG peak, the control strawberry sorbet was also extracted without the addition of the internal standard.

### 2.2. Treatments

#### 2.2.1. Ethics

Ethics approval for this study was granted by the Human Ethics Committee of the University of Newcastle, NSW, Australia (H2008-0089) and informed written consent was obtained from all the participants prior to commencement of the study.

#### 2.2.2. Participants and Selection Criteria

Four participants (3 males and 1 female) completed the study. They were healthy and aged between 18–64 years of age. Participants were excluded from the study if they were on any medication, dietary supplement or functional food to lower cholesterol or triglycerides. Exclusion criteria also included the following: baseline triglyceride levels ≥4.0 mmol/L, a history of coronary heart disease, a body mass index of ≥35 kg/m^2^, uncontrolled resting hypertension (≥160/95 mmHg), any known active pulmonary, hematologic, hepatic, gastrointestinal, renal, pre-malignant or malignant disease, diabetic, thyroid dysfunction or any pathology values known to be abnormal based on internationally accepted guidelines [45] and previous similar research conducted in humans using EGCG supplementation [27,42,46].

#### 2.2.3. Study Clinics

The participants attended three clinics in total, one for each of the three EGCG delivery methods. On each clinic visit, the standard anthropometric measurements of height and weight were taken and their BMI was calculated. Resting blood pressure was determined using a mercury sphygmomanometer (Livingstone International, Roseberry, NSW, Australia) with participants in the sitting position; the first and fifth Korotkoff sounds were taken to represent the systolic (SBP) and diastolic blood pressure (DBP), respectively. Measurements were done in triplicate to the nearest 2 mmHg and the average value was determined to be the participants’ blood pressure [47].

On each of the three clinics, after an overnight fast of at least 10 h, the participants ingested 500 mg of EGCG given either as two capsules (2 × 250 mg) with 100 mL of water only (without any breakfast), two capsules (2 × 250 mg) provided with 50 g of Special K breakfast cereal (Kellogg’s, Pagewood, NSW, Australia) served with 200 mL of full cream milk or EGCG (500 mg) incorporated in 200 g of strawberry sorbet (Table 1). All treatments were ingested within 5 min and no additional food was taken for a further 4 h. All participants were provided with a lunch and a drink three hours after ingesting the EGCG, which consisted of a sandwich (bread, ham, cheese, tomato, lettuce) and 200 mL of orange juice.

### 2.3. Blood Collection and Handling

An intravenous catheter (BD, North Ryde, NSW, Australia) connected to a SmartSite^®^ needle-free valve (Altaris Cardinal Health, Sydney, NSW, Australia) was inserted in the median cubital vein of the antecubital fossa of one of the participants arms and it was left in for 8 h. Approximately 5 mL of blood was collected into lithium heparin tubes (BD, North Ryde, NSW, Australia) before the ingestion of the EGCG (500 mg) as capsules or strawberry sorbet followed by an additional six collections of blood 30 min, 1 h, 2 h, 3 h, 5 h and 8 h after the ingestion of EGCG was completed. Immediately after collection, the blood samples were stored on ice in the dark and within 60 min of blood collection, plasma was separated by centrifugation, aliquoted under red light and samples were stored at −84 °C until assayed.

### 2.4. Determination of Free EGCG in Plasma Samples

#### 2.4.1. Preparation and Extraction of EGCG from Plasma Samples

Thawed plasma samples (200 μL) were diluted with 200 μL of pH 2 VcEDTA solution containing 5% (w/v) KH_2_PO_4_; 20% (w/v) ascorbic acid and 0.1% (w/v) EDTA) to enhance the stability of the EGCG and 200 μL of 0.05% (w/v) (+)-catechin to serve as an internal standard. The extraction of the catechins was performed by adding 1 mL of ethyl acetate and vortexing for 5 min and the organic layer was then transferred into a new tube and placed in a heating block (Ratec Instruments; Boronia; VIC; Australia) set at 50 °C and evaporated to dryness under a stream of nitrogen. The dried samples were re-dissolved in 20% (w/v) ascorbic acid pH 2 in 15% (*v*/*v*) acetonitrile and sequentially injected onto the HPLC. An external standard curve was prepared with pooled plasma samples spiked with known (+)-catechin and EGCG (15.6–1000 ng/mL) concentrations.

#### 2.4.2. Equipment and Chromatographic Conditions

The HPLC analysis was performed using a Finnigan Surveyor System (Thermo Fisher Electron Corporation, Sydney, NSW, Australia) equipped with a quaternary pump (Surveyor LC pump 1.4) and autosampler (Surveyor AS1.4) set at 4 °C and fitted with a 100 μL sample injection loop. Separation was performed using an analytical Prodigy ODS(3) 250 × 4.6 mm 5 μ column, protected by a guard column (Phenomenex, Pennant Hills, NSW, Australia) with the oven set at 26 °C. The elution times for (+)-catechin and EGCG were monitored using a Surveyor Photo-Diode array detector (Surveyor PDA 1.4) and UV-VIS absorption spectra was acquired over the range of 200–500 nm. The outlet of the PDA detector was directly connected to a mass spectrometer (Surveyor MSQ Plus 1.4 SUR 1). The full portion of the effluent was delivered into the ion source of the electrospray ionisation mass spectrometer (ESI-MS).

The mobile phases consisted of (A) 0.2% (*v*/*v*) formic acid (pH 2) and (B) acetonitrile. The auto-injector and needle wash solution consisted of acetonitrile:DI water (50:50 *v*/*v*). All solvents were filtered through a 0.45 μm Millipore cellulose filter (Millipore Australia, North Ryde, NSW, Australia) and degassed just prior to use.

The system was run at a flow rate of 1 mL/min with 87.5% mobile phase A and 12.5% of mobile phase B for the first 10 min after each injection. Then, for the following 30 min, the system was switched to a linear gradient concentration increase of mobile phase B to 25%. Mobile phase B was held for an additional 10 min at this concentration (25%) and then gradually decreased to 12.5% over the next 10 min. For the following 20 min, mobile phase A and B were run as described for the starting conditions (87.5% A and 12.5% B) to allow the column to re-equilibrate before the injection of the next sample.

The signals from the detectors (PDA and MS) were recorded and analyzed using the ExcaliburTM (v1.4 SR1) software (Thermo Fisher Electron Corporation, Sydney, Australia) installed on the computer assigned as a remote control operating system.

#### 2.4.3. Tuning and Setting Parameters of the ESI-MS

The tuning of the ESI-MS was optimised after the injection of 1 μg/mL pure EGCG or (+)-catechin in 0.2% formic acid (pH 2): acetonitrile (87.5:12.5 v/v) at a flow rate of 1 mL/min representing the chromatographic conditions prior to injection into the MS. The ESI probe temperature was set to 629 °C, the cone voltage to 75 V and the needle source voltage to 4 kV. Nitrogen was used as the nebulising gas at a flow rate of 50 L/h in order to create a fine spray of sample/solvent droplets.

From the optimisation procedure it was determined that the (+)-catechin would elute in the time range between 9 and 13 min and EGCG between 19 and 26 min. For subsequent analyses, the MS was operated in the selective ion monitoring mode (SIM) with negative polarity to detect the IS and EGCG as they eluted from the HPLC column.

Samples from pooled plasma spiked with 500 ng/mL EGCG were also used as a quality control for the plasma storage, extraction and assay system. This showed that the EGCG was stable during, storage, extraction and handling and that the extraction/analysis system was very robust; 98% ± 2% of the EGCG was recovered from the spiked plasma and the intra- and inter-assay variations were 2.2% and 3.7%, respectively.

### 2.5. Statistical Analysis

Pharmacokinetic analysis was performed in accordance with current industry guidance for orally administered pharmaceutical products [45]. The maximum concentration of EGCG from time 0 to 8 h was defined as C_max_, with T_max_ being the time required to reach the C_max_. The concentration of plasma EGCG at the end of the dosing interval was defined as C_min_ and the mean concentration during the dosing interval was defined as C_av_. The degree of fluctuation (DF) was also determined based on the formula (C_max_ − C_min_)/C_av_ while the swing of plasma EGCG was determined using (C_max_ − C_min_)/C_min_. The plasma EGCG elimination half-life (T_1/2_) was calculated based on the formula T_1/2_ = 0.693/Ke where Ke is the slope of the logarithmically transformed (ln) linear regression of the plasma EGCG concentrations [24,27]. The area under the curve (AUC_0–8_) analysis was determined using the linear trapezoidal rule from 0–8 h.

The statistical analysis of the pharmacokinetic variables and the anthropometric measurements was performed using the Statistical Package for Social Sciences (PASW Statistics 17) (SPSS Inc., Chicago, IL, USA). The one-way ANOVA and the Bonferonni *post-hoc* test were used to determine differences between the mean values of the anthropometric and pharmacokinetic variables obtained for the three EGCG delivery methods. The threshold for all statistical significances was set at *p* < 0.05 level. All pharmacokinetic data was calculated and presented in accordance with internationally accepted and standardized methods [45,48]. The correlations were also analyzed using linear regression analysis of the PASW Statistics 17.

## 3. Results

### 3.1. Recovery of EGCG in Strawberry Sorbet

The preparation of the strawberry sorbet exposed EGCG to conditions, such as solubilisation in an aqueous media, heating and exposure to light, which could have affected its stability and possibly have led to its degradation in the food matrix [29,31,33,34], Therefore, in order to ascertain that the amount of EGCG added in the sorbet was the amount that the participants would be receiving, EGCG was extracted from the strawberry sorbet after it had been prepared and frozen for at least one week at −20 °C.

**Figure 1:**
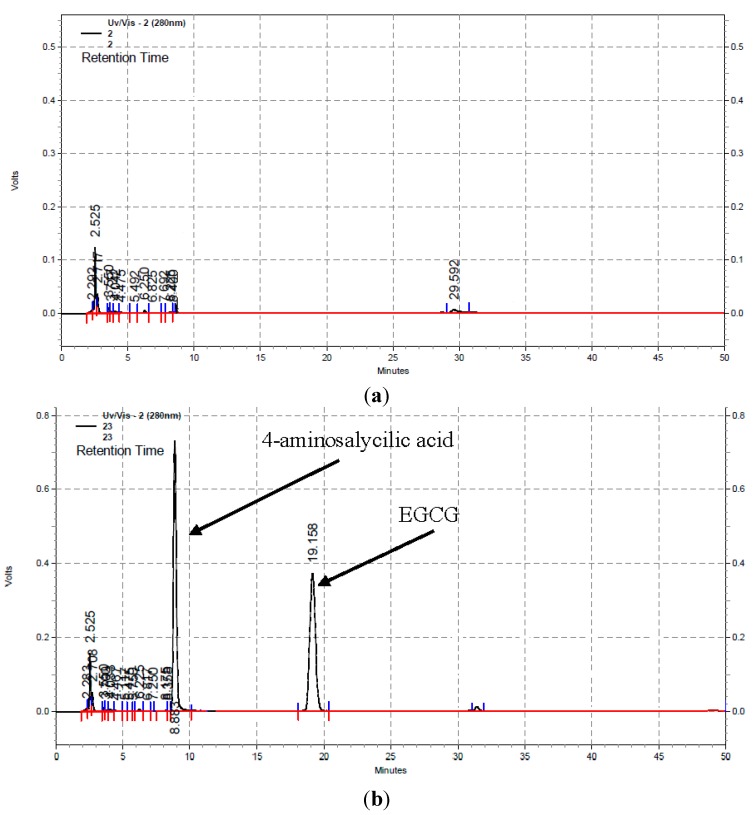
Typical HPLC-UV chromatograms (280 nm) of extracts from strawberry sorbet without (**a**) and with (**b**) the addition of EGCG (2.5 mg/g) and 4-aminosalycilic acid acting as internal standard (100 mmol/L).

Importantly, there was no trace of EGCG or the internal standard detected on the HPLC chromatogram of the extract from the control sorbet sample and there were no other interfering peaks (Figure 1); on the chromatogram obtained for the control sample (Figure 1a) there was no trace of any peaks at the respective retention times of the peaks for the internal standard and EGCG, as indicated in Figure 1b for a sample of the strawberry sorbet prepared with added EGCG (Table 1) and extracted after the addition of the internal standard, 4-aminosalycilic acid.

The amount of EGCG extracted was measured and the recovery of EGCG from the strawberry sorbet was expressed as a percentage of the EGCG originally added during the preparation of the strawberry sorbet. The results (Table 2) demonstrated that more than 97% of the EGCG originally added to the strawberry sorbet was extracted and therefore, it was ascertained that the amount of EGCG that the participants would be receiving was very close to the amount of EGCG added in the sorbet (Table 1).

**Table 2:** Recovery of EGCG from the strawberry sorbet.

*n*	EGCG Added to Strawberry Sorbet (mg/g)	EGCG Extracted from Strawberry Sorbet (mg/g) ^†^	EGCG Recovery (%) ^†^
5	2.50	2.44 ± 0.03	97.4 ± 1.3

^†^ Values represent the Mean ± SD for five different sorbet containers.

### 3.2. Measurements of EGCG in Human Plasma

The methods used for the HPLC analysis of individual catechins in tea samples commonly use UV detection. However, the amounts observable in plasma are well below the detection limits of UV detectors. The use of a MS as a detector for HPLC offers greater detection sensitivity and also greater specificity for the measurement of EGCG in biological fluids such as plasma. Therefore, the HPLC-ESI-MS analysis system was used to measure EGCG in human plasma but it first needed to be validated for specificity and sensitivity. Furthermore, (+)-catechin was used as the internal standard instead of 4-aminosalycilic acid; because its structure is closer to EGCG than 4-aminosalycilic acid, (+)-catechin was more detectable at the conditions for which the ESI probe was optimally tuned to detect EGCG: probe temperature at 629 °C, cone voltage at 75 V and needle source voltage to 4 kV.

**Figure 2:**
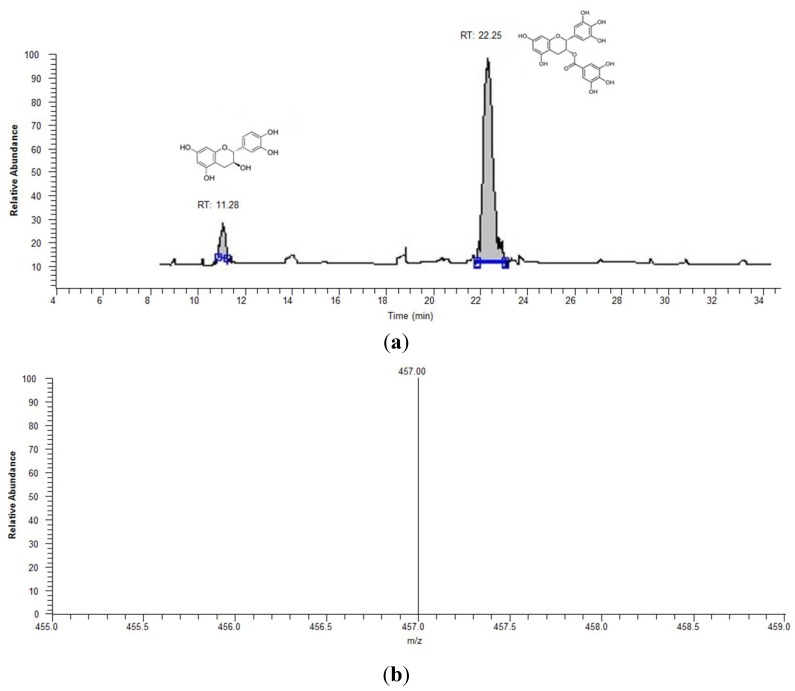
Typical chromatogram of the internal standard, (+)-catechin (100 ng/mL) and EGCG (500 ng/mL) analyzed by HPLC and ESI-MS set in negative polarity SIM mode. When the EGCG peak in (**a**) was selected the molecular ion for EGCG (molecular mass-1) was clearly seen at 457 m/z (**b**).

As seen in Figure 2, excellent chromatographic separation of the (+)-catechin (11.28 min), acting as the internal standard, and EGCG (22.25 min), was achieved without any interference from other plasma constituents. Therefore, using the ESI-MS, set in SIM mode, as the detector gave a clean HPLC chromatogram, which allowed clear identification and quantification of EGCG in the human plasma samples.

**Figure 3:**
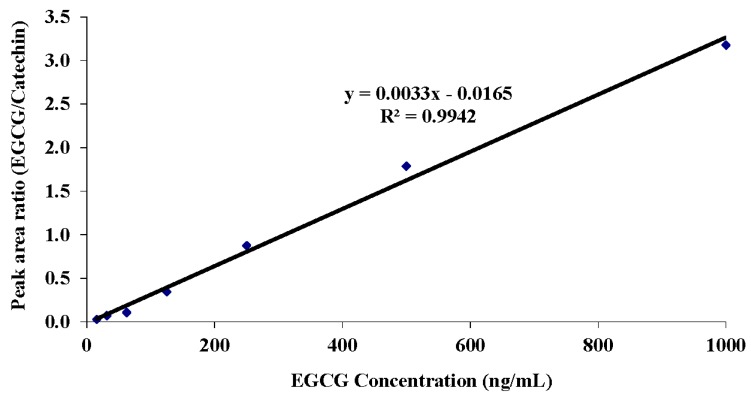
Calibration curve for EGCG spiked into human plasma at increasing concentrations and detected using the optimised HPLC ESI-MS system.

To determine the limit of quantification (LOQ), the limit of detection (LOD) and the linearity of the response, EGCG was added to plasma at seven different concentrations in quintuplicates (Table 3) and measured using the HPLC and ESI-MS system. The LOQ was evaluated as the EGCG concentration that resulted in a coefficient of variation (CV) of 5% or less; the results indicated that, using this system of extraction and detection, the lowest measureable concentration of EGCG in terms of precision was 31.25 ng/mL, with a CV of 4.78%. The LOD was identified to be lower than the LOQ at 15.63 ng/mL EGCG. Furthermore, the response, shown in Figure 3, was clearly very linear over the range of 15.6 to 1000 ng/mL for EGCG.

**Table 3:** Mean, standard deviation and coefficient of variation for seven concentrations of EGCG spiked into human plasma and detected using the optimised HPLC electrospray ionisation mass spectrometer (ESI-MS) system.

EGCG Concentration (ng/mL)	*n*	Mean Peak Area Ratio (EGCG/Catechin)	SD Peak Area Ratio	CV (%)
1000	5	3.18	0.004	1.21
500	5	1.79	0.041	2.32
250	5	0.88	0.023	2.67
125	5	0.35	0.014	4.01
62.5	5	0.11	0.003	2.82
31.25 ^†^	5	0.07	0.004	4.78
15.63 *	5	0.03	0.003	8.77

^†^ LOQ—the lowest concentration of EGCG with a CV < 5%; * LOD—the lowest detectable concentration of EGCG; *n*—the number of replicates.

### 3.3. Participants’ Anthropometric and Blood Pressure Data

Four participants, three males and 1 female with an average age of 31.25 ± 9.54 years, completed the study. Due to the limited number of participants, the results (Table 4) are presented indiscriminate of gender. The participants’ anthropometric data, standing height and weight, and their systolic and diastolic blood pressures were measured at the start of each clinic prior the ingestion of product containing EGCG.

The subjects’ measurements remained stable over the period of the study. Using the one-way ANOVA and the Bonferonni *post-hoc* test, it was found that there was no significant difference in BMI (*p* = 0.999), systolic blood pressure (*p* = 0.998), or diastolic blood pressure (*p* = 0.999) between the three clinic visits (Table 4).

**Table 4:** The anthropometric, BMI and BP results for the four participants at each clinic visit.

	Clinic 1	Clinic 2	Clinic 3
Weight (kg)	70.5 ± 10.7	70.7 ± 10.8	70.5 ± 10.8
Height (m)	1.75 ± 0.08	1.75 ± 0.08	1.75 ± 0.08
BMI (kg/m^2^)	23.1 ± 3.5	23.2 ± 3.6	23.1 ± 3.5
SBP (mmHg)	112.5 ± 5.0	115.3 ± 6.1	112.5 ± 5.0
DBP (mmHg)	85.0 ± 4.1	85.5 ± 3.3	82.5 ± 5

BMI—Body Mass Index; SBP—Systolic Blood Pressure; DBP—Diastolic Blood Pressure.

### 3.4. Plasma EGCG Concentration-Time Results

**Figure 4:**
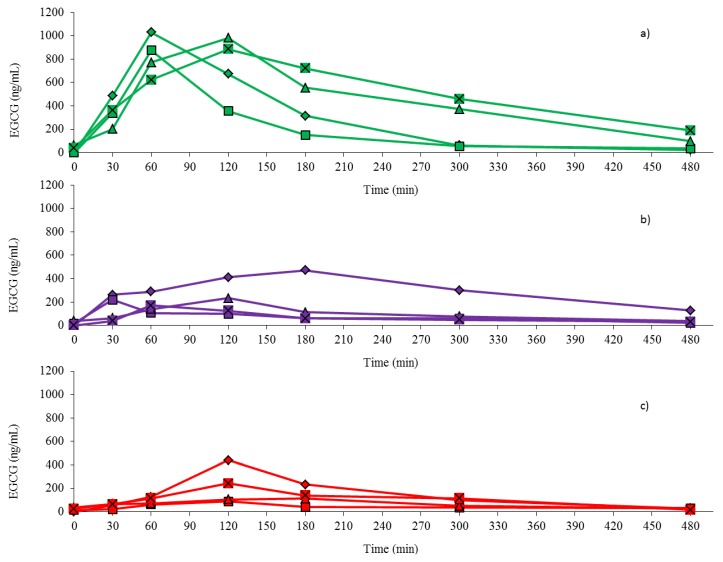
Plasma EGCG concentration-time curves for each of the four individual participants for the three different methods of EGCG oral delivery: (**a**) capsules without breakfast; (**b**) capsules with breakfast; and (**c**) strawberry sorbet.

**Figure 5:**
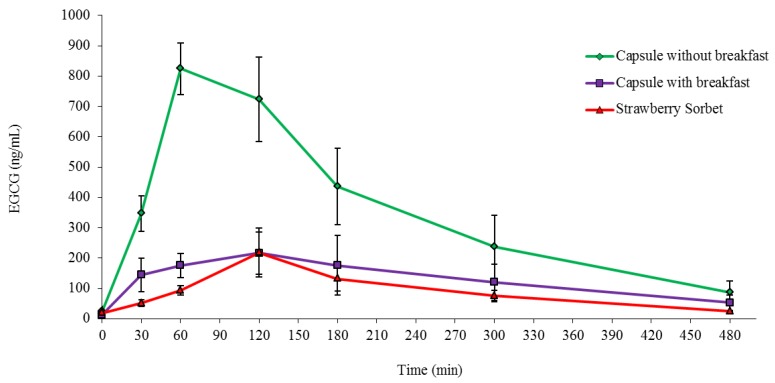
The mean plasma EGCG concentration-time curves for the three different methods of oral delivery: capsules without breakfast (green), capsules with breakfast (purple) and strawberry sorbet (red). The values are means ± the standard error of the means for the four participants (*n* = 4).

The plasma EGCG concentration was measured just before and over 8 h after the administration of 500 mg of EGCG delivered as two 250 mg capsules without breakfast, two 250 mg capsules with breakfast or 500 mg in 200 g of strawberry sorbet. The EGCG concentration curves over this period of time for each delivery method and for each participant (Figure 4) as well as the arithmetic mean EGCG concentration curves for each delivery method in all four participants (Figure 5) are presented.

Taking the two capsules of EGCG without breakfast resulted in a noticeably higher response than the other two treatments of the capsules of EGCG with breakfast or the strawberry sorbet containing EGCG (Figure 4). Moreover, the arithmetic mean concentration for plasma EGCG (Figure 5) reached a maximum value at 1 h (T_max_ = 60 min) for capsules taken without the breakfast, while the maximum concentrations for EGCG taken in capsules with breakfast and imbedded into the strawberry sorbet were reached at 2 h (T_max_ = 120 min) after the subjects ingested the EGCG.

### 3.5. Pharmacokinetic Parameters of Plasma EGCG

After the one-way ANOVA analysis and the Bonferonni *post-hoc* test it was noted that the AUC_0–8_ for EGCG taken as capsules without breakfast (Table 5) was significantly higher than the AUC_0–8_ for EGCG taken as capsules with breakfast (*p* = 0.044) and the AUC_0–8_ for EGCG taken incorporated in strawberry sorbet (*p* = 0.019) t . However, the difference between the AUC_0–8_ for EGCG taken as capsules with breakfast and the AUC_0–8_ for EGCG taken in the strawberry sorbet was not statistically significant (*p* = 1.000) (Table 5).

**Table 5:** Plasma kinetic parameters for EGCG after the three different methods of ingestion.

	EGCG Capsules Without Breakfast	EGCG Capsules With Breakfast	EGCG in Strawberry Sorbet
AUC_0–8_ (μg/mL/8 h)	173.8 ± 67.6 *	64.1 ± 53.7	44.5 ± 22.9
C_max_ (ng/mL)	824.2 ± 75.1 **	231.8 ± 134.3	218.0 ± 160.0
C_av_ (ng/mL)	382.6 ± 92.5 *	92.0 ± 46.0	87.6 ± 43.0
C_min_ (ng/mL)	86.9 ± 75.2	49.3 ± 24.6	25.0 ± 5.8
T_1/2_ (min)	78.6 ± 94.2	136.2 ± 85.2	228.6 ± 97.2
T_max_ (min)	60 ± 34.6 **	120 ± 34.6	120 ± 34.6
DF	1.93 ± 0.77	1.39 ± 0.46	2.20 ± 0.76
Swing	8.49 ± 17.19	3.36 ± 3.04	7.70 ± 6.61

Values are means ± standard deviations for the four participants (*n* = 4). * Value is significantly different from the other values in the row at the level of *p* < 0.05. ** Value is significantly different from the other values in the row at the level of *p* < 0.001. **AUC_0–8_**—area under the curve from 0 to 8 h; **C_max_**—maximum concentration; **C_av_**—Average concentration; **C_min_**—minimum concentration (at the end of the treatment); **DF**—degree of fluctuation; **T_max_**—time required to reach the maximal concentration; **T_1/2_**—half-life.

The C_max_ of EGCG in plasma (Table 5) after the ingestion of EGCG as capsules without breakfast was also significantly higher than when EGCG was taken as capsules with breakfast (*p* < 0.001) and higher than when it was taken in strawberry sorbet (*p* < 0.001). However, there was no significant difference in the C_max_ between the EGCG taken as capsules with breakfast and EGCG taken in strawberry sorbet (*p* = 1.000) (Table 5).

The C_av_ of EGCG in plasma for the eight hours after ingestion was also significantly higher for EGCG taken as capsules without breakfast (Table 5) than when taken as capsules with breakfast (*p* = 0.004) or taken in strawberry sorbet (*p* = 0.002). However, there was no significant difference between EGCG capsules taken with breakfast and taken in strawberry sorbet (*p* = 1.000) (Table 5).

From Table 5, it can also be noted that the C_min_, achieved at the end of the 8 h of monitoring time, the T_max_, the T_1/2_, the DF and swing for EGCG were not significantly different between the three treatment groups (*p* > 0.05).

## 4. Discussion

The results for the concentration of EGCG in plasma over the 8 h period after ingestion (Figure 4 and Figure 5, Table 5) clearly revealed that EGCG taken in capsule form by itself without food gave higher plasma values; the AUC was 2.7 times higher than when EGCG was taken in capsules with a light breakfast (*p* = 0.044) and 3.9 times higher than when EGCG was imbedded in the strawberry sorbet (*p* = 0.019). Furthermore, there was no significant difference in the AUC values (Table 5) between the EGCG taken as capsules with a light breakfast or taken in the strawberry sorbet (*p* = 1.000). This pattern was also observed when the values for C_max_ and C_av_ were compared between the three ingestion conditions (Table 5). Therefore, ingesting food at the same time as EGCG whether it was imbedded or not in food, substantially inhibited the absorption of the catechin. As with some types of medications that are affected by food, it appears that EGCG should be taken without food in order to maximise its intestinal absorption.

The concentrations of EGCG measured in the plasma after its ingestion in the present study are consistent with those of the only previous study that used similar doses of administered pure EGCG [27]. When 500 mg of EGCG was given in capsule form without food in the present study, the plasma EGCG concentration curve (Figure 4 and Figure 5) was very similar to the plasma EGCG values obtained when a similar dose of 400 mg EGCG was given without food to humans by Ullmann *et al.* [27]. Furthermore, the C_max_ and C_av_ values in the present study, 824 ng/mL and 383 ng/mL, respectively (Table 5), were very comparable to the C_max_ and C_av_ values, 862 ng/mL, and 568 ng/mL, respectively, observed in the Ullmann *et al.* [27] study.

The results in the present study are also very consistent with the previous findings of Chow *et al.* [42], who showed that food intake also clearly interfered with the systemic absorption of EGCG when it was given as part of a GT extract. Similar to the present study (Table 5), Chow *et al.* [42] also reported markedly higher plasma EGCG AUC (3.5 times higher) and C_max_ (5.7 times higher) values when the catechin extract was taken without food compared to when it was taken with a standardised breakfast consisting of muffins.

With varied food types, such as muffins [49], a breakfast cereal plus full cream milk and a strawberry sorbet (Figure 4 and Figure 5, Table 5), all decreasing the plasma EGCG concentration measured after ingestion, it is evident that food is an important factor, which can affect the intestinal absorption and systemic levels of orally administered EGCG, whether it is in a GT extract or in pure form. However, it is not known how food gives rise to this effect or which particular food component plays a role in decreasing the absorption of EGCG.

This is also not entirely surprising because it is well known that the absorption of some pharmaceutical medications is decreased when they are taken with various food products. From what is known about the interactions of food with medications and plant bioactives [50,51], several factors may influence the bioavailability of EGCG. These factors can be divided into three broad categories: (1) the effect of the vehicle in which the EGCG was administered; (2) the effect of the biological fluids on EGCG prior to it reaching its absorption site and; (3) effects on EGCG due to physiological responses to the ingested food products.

Relative to the first category—the effect of the vehicle in which the EGCG was administered—incorporating EGCG in a food product like a strawberry sorbet could have been expected to improve the absorption of EGCG compared to taking the catechin in capsule form with a breakfast. It is well known that EGCG is relatively unstable and susceptible to degradation [29] at high temperatures [31] and at pH values above four [34]. Therefore, imbedding the EGCG in a food product like strawberry sorbet, which is stored at −20 °C and has a pH below 4, may have increased the stability of the EGCG. This was supported by the finding that the EGCG was very stable in this food product, as evidenced by the very high percentage (over 97%) observed for its recovery from the strawberry sorbet after storage at −20 °C (Table 1). This finding indicated that the method used to prepare the strawberry sorbet and its acidic environment preserved the EGCG extremely well [37] and therefore, the amount of chemically intact EGCG (500 mg) ingested in the 200 g of sorbet given to the human volunteers was the intended amount.

Evidently however, the fact that the EGCG was chemically intact and stable in the strawberry sorbet did not improve its bioavailability (Figure 4 and Figure 5, Table 5). Obviously, similar to the breakfast cereal and the full cream milk in the present study and the muffins in the study by Chow *et al.* [42], the presence of food components in the strawberry sorbet must have played a role in reducing the bioavailability of EGCG and resulted in a significantly lower AUC for EGCG than when it was taken on its own without food.

Relative to the second category—the effect of the biological fluids on EGCG prior to it reaching its absorption site—the principal biological fluids EGCG would come into contact with, are the gastric juice and the pancreatic/biliary juices. Saliva was unlikely to be a factor, as the EGCG capsules taken with the light breakfast were very quickly swallowed with water and therefore, the EGCG was unlikely to have been exposed to much saliva.

In the stomach, little degradation of EGCG is expected to occur because of the acidic nature of the gastric secretions. In effect, as shown by Record and Lane [52], EGCG was stable in acidic solutions (pH < 3) made up to mimic those found in the fasting stomach environment (pH 1.5–2) [53]. However, any increase in pH caused by protein or any other component of food could have led to an accelerated degradation of EGCG and reduced its bioavailability [27,42,54].

In the small intestine, the acidic chyme that is pushed down from the stomach is quickly neutralised by the bicarbonate solution secreted by the pancreas into the duodenum [50,55]. This is where most of the EGCG is expected to be lost. As shown by Record and Lane [52], EGCG is particularly unstable under conditions which mimic digestion fluids in the small intestines, with only 1% of the EGCG still measurable after an hour incubation.

Hirun and Roach [37] and Green *et al.* [38] showed that the stability of EGCG under these simulated digestion conditions increased when EGCG was in a strawberry sorbet or mixed with fruit juices, respectively. However, the current studies suggest that this is unlikely to happen under the true small intestine conditions in humans, as imbedding the EGCG in a strawberry sorbet in the present study did not lead to any improvement in absorption compared to taking the EGCG in capsules with the light breakfast. Most likely, the amount of bicarbonate solution secreted by the pancreas into the duodenum was enough to fully neutralise any acidity brought down to the small bowel by the strawberry sorbet.

Evidently, the possibility that the acidic nature of the strawberry sorbet could keep the pH of the small intestine less basic either did not occur or was not a factor in the bioavailability of the EGCG it contained. Clearly, other ways of protecting the EGCG from the basic pH in the small intestines are needed. Several studies have reported different methods of preserving the EGCG such as encapsulation using oil-in-water sub-micrometer emulsions [56], lyposomes [57] and protein/polyphenols microparticles [58], but whether they can preserve the EGCG from degradation in the small intestine and increase its bioavailability remains to be determined.

It is possible that taking the capsules without food may have allowed the EGCG to survive longer in the small intestines and therefore, enhance its chances of being absorbed, because it did not elicit strong responses from the stomach and the pancreas. The EGCG capsules by themselves could be expected to have caused the stomach to produce much less acidic chyme than when the EGCG was taken along with food, either in the form of the strawberry sorbet or the breakfast cereal with full cream milk. Consequently, this could have elicited a less strong response from the pancreas to secrete bicarbonate solution to neutralise the chyme coming down from the stomach.

Relative to the third category—effects on EGCG due to physiological responses to the ingested food products—the small intestine is the primary absorption site for EGCG and the rate at which EGCG is presented into the upper portion of the small intestine and travels down to its absorption site can determine the bioavailability of the catechin. It is known that the ingestion of food can delay the rate of gastric emptying [51,53] and that the rate of gastric emptying is one of the most important factors known to influence the absorption rate of orally administered pharmaceuticals from the gastrointestinal tract [51].

In concurrence with this, EGCG taken with the breakfast and in the strawberry sorbet showed a delay in time it took to reach its maximum concentration in plasma (T_max_ in Table 5) compared to EGCG taken without food. Therefore, a slower gastric emptying in the presence of food most likely prolonged the time needed for EGCG to travel into the upper portion of small intestine. However, given that the bioavailability of the EGCG was much lower when it was taken with food, some of the extra time is likely to have been spent transiting through the small intestine where exposure to a high pH for longer could have contributed to a greater degradation.

Another possibility, which could have explained the higher plasma EGCG concentrations observed when the catechin was taken without food, was an increased clearance rate of EGCG from the plasma when it was taken with food. However, the mean elimination half-life (T_1/2_ in Table 5) was not significantly different between the three ingestion methods (*p* > 0.05). Therefore, the ingestion of EGCG with food or incorporated in a food did not appear to significantly influence the clearance rate of free EGCG from the systemic circulation once it was absorbed.

The main limitation of this study is the low number of participants (*n* = 4) and it would be useful to repeat the study with a higher number of subjects. Nevertheless, despite the low numbers, the inhibitory effect of food on the systemic absorption of the pure EGCG was strong (3–4 times lower with food than without food) and unequivocal (statistically significant). The strength of the cross-over study design may have helped; with each participant acting as their own control in this design, the impact of inter-individual variation is minimised. Therefore, the results were unequivocal in revealing that the EGCG was best absorbed when consumed in capsule form without any food after the overnight fast, a finding which supports the results of Chow *et al*., the only previous study to study the effect of food on the systemic absorption of EGCG, although it was given as part of green tea extract and not as a pure catechin.

## 5. Conclusions

In conclusion, the systemic absorption was significantly higher for EGCG taken in capsules without food after an overnight fast than it was when it was taken in capsules with a light breakfast or imbedded in a strawberry sorbet. Therefore, based on these findings, ingesting EGCG with water on an empty stomach is the most appropriate method for the oral delivery of EGCG in future clinical trials where EGCG is to be investigated as a potential bioactive nutraceutical in humans.
